# Schizophrenia in Autistic People with Intellectual Disabilities. Treatment and Interventions

**DOI:** 10.1007/s10803-024-06286-6

**Published:** 2024-02-23

**Authors:** Trine Lise Bakken, Jane Margrete Askeland Hellerud, Arvid Nikolai Kildahl, Ann Magritt Solheim-Inderberg, Oddbjørn Hove, Sissel Berge Helverschou

**Affiliations:** 1 Oslo University Hospital, Oslo, Norway; 2 Helse Fonna Health Trust, Haugesund, Norway

**Keywords:** Intellectual disabilities, Autism spectrum disorders, Schizophrenia, Treatment, Interventions

## Abstract

Autistic people with intellectual disabilities appear to be at increased risk of schizophrenia. While current recommendations emphasize adapting interventions used for people with schizophrenia in general, few studies to date have investigated treatment of co-occurring schizophrenia in this specific population. To explore what interventions are provided to autistic people with intellectual disabilities and co-occurring schizophrenia in specialized mental health services, and to investigate whether changes in mental health symptoms and challenging behavior occurred during treatment. Using data from a longitudinal, national multicenter study, interventions provided to 26 autistic individuals with intellectual disabilities and co-occurring schizophrenia were explored. Symptoms were measured using the Psychopathology in Autism Checklist (PAC) and the Aberrant Behavior Checklist ABC) at referral (T1), at the end of treatment (T2), and at follow-up 12 months after T2 (T3). A broad range of interventions were provided to the participants, including inpatient admission, psychopharmacological treatment, various psychosocial interventions, and supportive interventions. Scores on the PAC and ABC were significantly lower at T2 than T1 for most scales, and no significant change was found from T2 to T3.Treatment of co-occurring schizophrenia appears feasible and effective in autistic people with intellectual disabilities.

## Introduction

Autistic individuals with intellectual disabilities appear to be at increased risk of schizophrenia (Korb & Hassiotis, 2022; Vaquerizo-Serrano et al., 2022; Lai et al., 2019; Zheng et al., 2018; Chisholm et al., 2016; Bakken et al., 2010). Treatment of schizophrenia in this population has been sparsely described (Bakken et al., 2023; Bakken, 2021). Schizophrenia spectrum disorders constitute serious mental illnesses involving distinct clusters of symptoms: positive (hallucinations, delusions, disorganized thinking/speech), negative (blunted affect, loss of motivation, loss of the ability to feel pleasure, apathy), and cognitive (particularly verbal memory and attention) (American Psychiatric Association, 2013). Among the psychotic disorders, schizophrenia is considered one of the most severe (American Psychiatric Association, 2013). Co-occurring schizophrenia in autistic people with intellectual disabilities has drawn increased attention in recent years (Marin et al., 2018; Upthegrove et al., 2018; Bakken et al., 2016). However, research appears to have focused primarily on prevalence and diagnostic assessment (Bakken et al., 2007, 2009, 2016, 2023; Rosen et al., 2018; Underwood et al., 2015; Helverschou et al., 2011), with treatment and intervention appearing to constitute a current knowledge gap.

Current treatment recommendations for schizophrenia in autistic people with intellectual disabilities include adaptation of treatment and interventions used for schizophrenia in non-autistic people without intellectual disabilities (Korb & Hassiotis, 2022; Deb et al., 2022b; Deb, Bertelli Deb et al., 2022a, b; Bakken, 2021; Chisholm et al., 2016; Strålin & Hetta, 2019; Bakken & Høidal, 2014; Bakken et al., 2007). In addition to adapting interventions to the patient’s current symptom load and phase of illness, they need to be adapted to the patient’s style of communication and autism-related difficulties, and also to their verbal, emotional, and cognitive functioning (Korb & Hassiotis, 2022; Deb, Bertelli Deb et al., 2022a, b; McPherson et al., 2020; Bakken & Høidal, 2014). Moreover, autistic people with intellectual disabilities may display atypical symptoms of schizophrenia, including challenging behaviors (Bakken et al., 2023; Kildahl et al., 2023a; Bakken, 2021). Thus, evaluating treatment may be as challenging as diagnosing schizophrenia in this population (Kildahl et al., 2023), and an adequate understanding of the specific individual’s symptoms therefore appears to be as important during treatment and follow-up, as during assessment.

Evidence-based treatment of schizophrenia involves biological, psychological, and social aspects; typically psychopharmacological treatment, psychosocial interventions such as psychotherapy and mental health nursing (a branch of nursing, developed during the institutional area, including a knowledge base on mental illnesses, and core mental health nursing skills, Nolan, 1998), community services, and follow-up over time (Ventriglio et al., 2020; Humphries et al., 2020; van Os & Kapur, 2009). Research on these interventions is sparse for people with co-occurring intellectual disabilities (Korb & Hassiotis, 2022), and even more sparse for individuals who are also autistic (Chisholm et al., 2016). However, Korb and Hassiotis (2022) recommend that treatment of psychotic disorders in people with intellectual disabilities makes use of the full range of psychosocial and psychopharmacological treatment interventions.

While high-level evidence on the efficacy of psychopharmacological treatment of schizophrenia in this population is lacking (Deb et al., 2022b; Korb & Hassiotis, 2022), following the general guidelines for treatment of schizophrenia is recommended (Humphries et al., 2020). Regarding psychosocial interventions, research is limited in autistic individuals with intellectual disabilities and co-occurring schizophrenia (Korb & Hassiotis, 2022; Chisholm et al., 2016). However, several psychosocial interventions have been described to have positive effects, for example, in case studies or small samples (Bakken et al., 2017; Bakken & Høidal, 2014). Regarding mental health nursing, the ability to establish joint attention with the patient, providing sufficiently adapted assistance with activities of daily living, and also responding meaningfully to the patient’s initiatives and providing emotional support (see also Sommerstad et al., 2021; Bakken et al., 2017; Kildahl et al., 2017). Regarding follow up in community settings, assertive community treatment (ACT) has been found to be feasible for individuals with intellectual disabilities and schizophrenia (Neijmeijer et al., 2018; Martin et al., 2005). Because of the risk of relapse, follow-up is crucial for patients with schizophrenia (Emsley et al., 2013).

To sum up, while using the range of psychopharmacological and psychosocial treatment options available has been recommended in autistic people with intellectual disabilities and co-occurring schizophrenia (Korb & Hassiotis, 2022), it remains unclear what interventions are provided to these patients. Moreover, while positive effects of these interventions have been described in case studies, for example, it is unclear to what degree they are associated with symptom relief in this specific population.

## Aims and Research Questions

The current study aims to explore what treatment interventions are provided to autistic individuals with intellectual disabilities diagnosed with co-occurring schizophrenia in specialized mental health services. In addition, the study aims to explore whether the treatment period is associated with changes in reported symptoms and behaviors using standardized checklists for this population. Consequently, we wanted to answer the following research questions:

1. What kind of interventions are provided to autistic people with intellectual disabilities diagnosed with co-occurring schizophrenia?
2. What are the reported changes in reported mental health symptoms and challenging behaviors in autistic people with intellectual disabilities treated for schizophrenia, at the end of treatment/discharge, and after one year?

## Methods

### Design and Setting

The current study uses data from a Norwegian, nationwide multi-center study, the AUP study (Autism, Intellectual Disability, Mental illness study) (Helverschou et al., 2021). Eight centers providing specialized mental health services for autistic people with ID participated in the study, collecting data between 2010 and 2020. The AUP-study is a longitudinal study, collecting data at three time points, using a standardized assessment protocol: before treatment (T1), after 12 months or end of treatment (T2), and after 24–27 months (T3). The current study uses data from all three time points. Preliminary results from the AUP study, including details concerning protocol and the involved centers are described in an earlier publication (Helverschou et al., 2021).

### Participants

Participants were diagnosed with co-occurring autism spectrum disorder (ASD) and intellectual disability according to the ICD-10 (World Health Organization, 1992), prior to inclusion (Kildahl et al., 2023a; Helverschou et al., 2021a). In addition, inclusion criteria included suspected mental health disorder or challenging behavior. The involved centers themselves selected participants from their respective health regions (Helverschou et al., 2021). No exclusion criteria were applied in the AUP study with regard to further co-occurring health problems or disabilities.

The current study includes all participants in the AUP study who were diagnosed with co-occurring schizophrenia during the course of the study: 26 participants aged 16–49 (*M* = 26.73, *SD* = 8.74), including 9 females (34.6%) and 17 (65.4%) males. Of these, 24 (92.3%) had been diagnosed with mild/moderate intellectual disabilities and 2 (7.7%) had been diagnosed with severe/profound intellectual disabilities. Assessment of schizophrenia in this specific sample, including symptom manifestations, has been described in a previous publication (Bakken et al., 2023).

### Measures

#### Interventions

Interventions were reported by the participating centers on a standardized form, including 27 possible interventions, where the responsible practitioner had to confirm or disconfirm whether each of these had been provided to the participant in question. The list was created especially for the AUP study and included different types of interventions frequently provided in mental health care for people with intellectual disabilities (see below). These included 20 patient-directed interventions (e.g., psychopharmacological treatment, psychotherapy, crisis management plan), and 5 supportive interventions (e.g., collaborative meetings, supervision/training of community caregivers). Interventions were registered continuously, and the centers submitted the intervention form at T3. However, the patient-directed interventions would all have been provided before T2.

#### Psychopathology in Autism Checklist (PAC)

The PAC is a proxy-rated screening checklist for mental health disorder developed for autistic people with intellectual disabilities (Helverschou et al., 2008, 2009). It includes 42 items scored on a 4-point Likert scale (1 = no problem, 2 = minor problem, 3 = moderate problem, 4 = severe problem). Scores are used to calculate five scale scores; psychosis (10 items), anxiety (6 items), depression (7 items), obsessive compulsive disorder (7 items) and a scale for general adjustment problems (12 items). Previous studies have found the PAC both to distinguish between mental disorder and core symptoms of ASD (Helverschou et al., 2008), and to distinguish between autistic individuals with intellectual disabilities according to whether they meet criteria for a co-occurring mental health disorder (Bakken et al., 2010; Helverschou et al., 2009; Helverschou, Ludvigsen, Helverschou et al., 2021a, b; Kildahl et al., 2023). To some extent, the PAC also appears to distinguish between autistic individuals with different mental disorders in this population (Dalhaug et al., 2022; Helverschou, Ludvigsen, Helverschou et al., 2021a, b). While cut-off values for screening have been identified (Helverschou et al., 2009), the PAC was used as a continuous measure in the current study, with higher scores reflecting severity of reported symptoms on each scale.

#### Aberrant Behavior Checklist (ABC)

The ABC (Aman et al., 2012; Aman & Singh, 1986; Aman et al., 1985), is a proxy-rated checklist for challenging behaviors in people with intellectual disabilities, and was originally developed to measure treatment effects in this population (Aman, 2012). It is a widely used checklist and has been used in numerous studies (Aman, 2012; Helverschou et al., 2020), with psychometric properties varying from satisfactory to excellent (Aman, 2012; Flynn et al., 2017; Helverschou et al., 2020). The ABC has shown good psychometric properties across different levels of intellectual disabilities (Aman, 2012; Flynn et al., 2017), including for its Norwegian version (Halvorsen et al., 2019). The ABC consists of 58 items scored on a 4-point Likert scale (0 = no problem, 1 = minor problem, 2 = moderate problem, 3 = severe problem). Scores are used to calculate five scale scores: irritability/agitation/crying (15 items), lethargy/social withdrawal (16 items), stereotypic behavior (7 items), hyperactivity/noncompliance (16 items), and inappropriate speech (4 items). The ABC is a dimensional measure, and higher scores on the respective scales reflect more severe levels of challenging behavior within these domains.

### Analyses

To determine the mean symptom load for each of the three time points: before treatment (T1), after treatment (T2) and at follow up (T3), we conducted a one-way repeated measures ANOVA for all PAC and ABC scale scores. Due to the small study sample, we chose to use the Greenhouse-Geisser adjustment when interpreting the results from the ANOVA (Blanca et al., 2023). Where the within-subjects effects were significant, we used pairwise comparison to identify the respective time points accounting for the significant change in symptom severity. The Bonferroni correction was used to correct for multiple comparisons.

Frequency analyses were conducted for reported treatment interventions. Only interventions provided to more than one patient were included. The very few missing values on the PAC (18/3276, 0.55%) and ABC (9/4524, 0.20%) were re-coded as a score of “no problem”. An alpha level of 0.05 was chosen for the statistical analysis due to the small sample size and the explorative nature of the current study. All data were analyzed using the Statistical Package for Social Science (SPSS) version 28.

### Ethical Considerations

The AUP multi-center study was approved by the Privacy Data Protection Supervisor (Local institutional review board) at Oslo University Hospital, Oslo, Norway, #2010/19,579. Due to participants’ limited capacities for consent, informed consent was obtained from their legal guardians for all participants. In addition, participants consented themselves whenever feasible. All data were anonymized prior to submission from each center.

## Results

### Frequency of Treatment Interventions

Psychopharmacological treatment was provided for 25 out of 26 participants (92.6%). For 22 (84.6%) this included at least one antipsychotic agent. Thirteen participants (50%) were prescribed antipsychotics only, whilst 9 (34.6%) participants were prescribed additional psychopharmacological treatment. Antidepressants (*n* = 7, 28%) were the most frequent in addition to antipsychotics, followed by antiepileptics (*n* = 2, 8%), see Table 1. One participant received an antidepressant only, and only one participant was prescribed three different psychopharmacological agents, an antipsychotic, an antidepressant, and an antiepileptic medication. More specific information was not available about types of medication and dosage.

**Table 1:** Psychopharmacological treatment of the patients with schizophrenia, *n* = 25

Age	Gender	Type of medication	Total agents
25	Male	AP, MS	2
49	Female	AP, AD	2
17	Male		0
26	Male	AP	1
31	Female	AD	1
17	Male		0
23	Male	AP, AD	2
33	Female	AP, AE	2
33	Male	AP	1
24	Male	AP	1
26	Female	AP, AD, AE	3
24	Male	AP	1
33	Female	AP	1
16	Female	AP	1
26	Female	AP	1
18	Female	AP	1
44	Male	AP	1
16	Male	AP, AD	2
35	Male	AP	1
20	Male	AP	1
42	Male	AP, AD	2
18	Male	AP	1
23	Female	AP, AD	2
26	Male	AP, SA	2
27	Male	AP, ANX	2

*Note* AP = Antipsychotic, AD = Antidepressant, ANX = Antianxiety medication, SA = Sleep agent, MS = Mood stabilizer, AE = Antiepileptic

As for other patient-directed treatment interventions, daily activities were adapted according to their schizophrenia for 24 (92.3%) participants. Differentiated intervention plans according to symptom load (*n* = 23, 88.5%), identification of warning signs (*n* = 22, 84.6%), and crisis management plans, adjustment of demands, and inpatient treatment (*n* = 23, 88.5%) were also frequently reported, see Table 2. Supportive interventions such as collaborative meetings, community staff supervision/training, and supervision of county level specialist mental health services were frequent, respectively provided to 26 (100%), 25 (96.2%), and 24 (93.2%) of the participants, see Table 3. Consultations with patients’ families were conducted in 18 (69.2%) of the 26 cases.

**Table 2:** Frequencies patient treatment interventions, *n* = 26

Intervention	n (%)
Psychopharmacological treatment	25 (96.2)
Activities adapted to diagnosis	24 (92.3)
Differentiated intervention plan	23 (88.5)
Warning signs identified	22 (84.6)
Crisis management plan	20 (76.9)
Inpatient treatment	20 (76.9)
Adjustment of demands	20 (76.9)
Increased focus on praise	17 (65.4)
Psychotherapy	13 (50)
Increased focus on autonomy	11 (42.3)
Daily schedule implemented	10 (38.5)
Other adjustments of environment	5 (19.2)
Behaviour contract applied	4 (15.4)
Multifamily group	2 (7.7)
Reward system implemented	2 (7.7)

**Table 3:** Frequencies supportive interventions, *n* = 26

Intervention	n (%)
Collaboration meetings	26 (100)
Community staff supervision/training	25 (96.2)
Supervision of county level health services	24 (92.3)
Conversation with patients family	18 (69.2)

#### Outcome Measures

For the PAC, the one-way repeated measures ANOVA across the three time points revealed significant reductions in scores on PAC psychosis (*p* < .001, *η*^*2*^_*p*_ = 0.314), PAC depression (*p* = .018, *η*^*2*^_*p*_ = 0.016), PAC anxiety (*p* = .013, *η*^*2*^_*p*_ = 0.161), and PAC general adjustment problems (*p* < .001, *η*^*2*^_*p*_ = 0.227), see Table 4. Larger effect sizes were observed for PAC psychosis and PAC general adjustment problems. No significant change was found for PAC OCD. Pairwise comparisons revealed that the significant decreases in scores were associated with the time from T1 to T2 for PAC psychosis, PAC depression, and PAC general adjustment problems, and with the time from T1 to T3 for PAC anxiety, see Table 5. No significant changes were found for any PAC scale from T2 to T3.

**Table 4:** Repeated measures ANOVA by PAC and ABC subscales, mean sum scores at the three time points and within-subjects effects, *n* = 26

		T1		T2		T3					
Scale	Min-max	M	SD	M	SD	M	SD	df	F	p	η^2^_*p*_
PAC general adjustment problems	12–48	28.85	7.12	24.00	5.58	23.85	7.47	(2, 50)	9.56	< 0.001**	**0.277**
PAC psychosis	10–40	24.19	5.93	18.27	5.44	19.31	7.41	(2, 50)	11.43	< 0.001**	**0.314**
PAC obsessive compulsive disorder	7–28	12.23	4.64	11.31	4.29	10.77	4.14	(2, 50)	1.78	0.180	0.067
PAC depression	7–28	16.96	5.05	14.46	4.37	14.31	5.16	(2, 50)	4.62	0.018*	0.016
PAC anxiety	6–24	11.96	3.59	11.08	2.87	9.73	3.12	(2, 50)	4.80	0.013*	**0.161**
ABC irritability	0–45	17.12	11.63	10.38	9.07	11.92	10.94	(2, 50)	5.69	0.007*	**0.185**
ABC lethargy	0–48	16.35	9.20	12.58	7.69	14.46	10.41	(2, 50)	1.95	0.157	0.072
ABC stereotypy	0–21	4.92	4.62	3.24	3.60	2.48	2.43	(2, 48)	5.06	0.012	**0.174**
ABC hyperactivity	0–48	19.27	11.05	9.46	6.24	9.54	9.06	(2, 50)	14.46	< 0.001**	**0.366**
ABC inappropriate Speech	0–12	5.23	3.40	2.65	2.34	3.65	3.10	(2, 50)	8.89	< 0.001**	**0.262**

*p < .05, **p < .001, larger effect sizes in bold. *Note* Only corrected p-values, using the Greenhouse-Geisser adjustment are presented above

**Table 5:** Pairwise comparisons of estimated marginal means on PAC and ABC subscales, Bonferroni correction for multiple comparisons

Measure		Time					
		T1-T2		T1-T3		T2-T3	
	Min-max	Mean difference	p	Mean difference	p	Mean difference	p
**PAC**							
General adjustment problems	12–48	4.846	< 0.001**	5.000	0.003*	0.154	1.000
Psychosis	10–40	5.923	< 0.001**	4.885	0.004*	-1.038	1.000
Obsessive compulsive disorder	7–28	0.923	0.637	1.462	0.213	0.538	1.000
Depression	7–28	2.500	0.017*	2.654	0.032*	0.154	1.000
Anxiety	6–24	0.885	0.675	2.231	0.025*	1.346	0.180
**ABC**							
Irritability	0–45	6.731	0.006*	5.192	0.049*	-1.538	1.000
Lethargy	0–48	3.769	0.087	1.885	1.000	-1.885	1.000
Stereotypy	0–21	1.680	0.150	2.440	0.027*	0.760	0.802
Hyperactivity	0–48	9.080	< 0.001**	9.731	0.001*	− 0.077	1.000
Inappropriate speech	0–12	2.577	< 0.001**	1.577	0.051	-1.000	0.437

*p < .05, **p < .001

For the ABC, the one-way repeated measures ANOVA across the three time points revealed significant reductions for ABC irritability/agitation/crying (*p* = .007, *η*^*2*^_*p*_ = 0.185), ABC stereotypical behavior (*p* = .012, *η*^*2*^_*p*_ = 0.174), ABC hyperactivity/noncompliance (*p* < .001, *η*^*2*^_*p*_ = 0.366), and ABC inappropriate speech (*p* < .001, *η*^*2*^_*p*_ = 0.262), see Table 4. Larger effect sizes were observed for ABC hyperactivity/noncompliance and ABC inappropriate speech. No significant change was found for ABC lethargy/social withdrawal. Pairwise comparisons revealed that the significant decreases in scores were associated with the time from T1 to T2 for ABC irritability/agitation/crying, ABC hyperactivity/noncompliance, and ABC inappropriate speech, and with the time from T1 to T3 for ABC stereotypic behavior, see Table 5. No significant change was found for any ABC scale from T2 to T3.

## Discussion

### Main Findings

A relatively broad spectrum of interventions was provided, which thus appear to promising for future research of treatment of schizophrenia in autistic individuals with intellectual disabilities. Furthermore, participants scored significantly lower on scales for mental health symptoms and challenging behavior at discharge/end of treatment, indicating that treatment of co-occurring schizophrenia is effective in autistic people with intellectual disabilities. These improvements appeared to remain stable a year later.

### The Interventions

Twenty-three of the 26 participants were admitted to inpatient units. This finding is not unexpected, as loss of adaptive functioning is common in schizophrenia, and the range of recommended interventions may be challenging to provide in outpatient settings during acute phases. In a review of characteristics of patients with intellectual disabilities admitted to inpatient wards, Bakken and Martinsen (2013) found that psychosis was the most common diagnosis, highlighting both the severity of these conditions and the need for provision of appropriate inpatient treatment for autistic people with intellectual disabilities and co-occurring schizophrenia.

The broad spectrum of interventions delivered provides further evidence that the range of psychopharmacological and psychosocial treatment interventions developed for schizophrenia appear to be useful also for autistic patients with intellectual disability. While information is lacking about how these interventions were adapted, they were delivered in specialized mental health services, indicating that they can be adapted by professionals with specific knowledge concerning mental health in autistic individuals with intellectual disabilities.

Psychopharmacological treatment was the only intervention provided to all participants, with 25 of the 26 participants being prescribed antipsychotics. This is in line with guidelines for treatment of schizophrenia (Korb & Hassiotis, 2022; Humphries et al., 2020), and in line with previous research indicating that autistic people with co-occurring psychosis are as likely to be prescribed antipsychotics as other patients with psychosis (Treise et al., 2021; Bakken & Høidal, 2019; Hui et al., 2018). Antipsychotic medication is found to be effective mostly regarding positive symptoms, while less effective towards negative symptoms (ibid.). Adjustments of medication regimes are necessary relating to the cognitive, social, and communicative functioning of autistic individuals with intellectual disabilities (Humphries et al., 2020). They may be particularly sensitive to side effects, such as tardive dyskinesia (Fodstad et al., 2010) and others (Downs et al., 2017; McPheeters et al., 2011). Systematic monitoring of potential side effects is important because these individuals may have difficulties reporting such effects (Jobski et al., 2017; Tveter et al., 2016; Fodstad et al., 2010). Moreover, these individuals may not understand why they feel different when receiving psychopharmacological treatment, indicating that longer intervals between dosage increases may be helpful to help the patient adjust during initiation of treatment (Rysstad et al., 2022). The reduction of the PAC scores regarding both psychotic symptoms and anxiety symptoms from T1 to T3 support both clinical experiences of treatment of psychotic conditions in autistic people with ID (Bakken et al., 2007), and recommendations of combination treatment of schizophrenia in adults in the general population (Kuipers et al., 2014).

A combination of medication and psychosocial interventions were provided for all participants in the present sample. In general, antipsychotic medication will not be a sole solution for persons suffering from schizophrenia (van Os & Kapur, 2009). This findings is in line with previous case studies / studies with small samples on schizophrenia treatment for autistic adults with intellectual disabilities (Sommerstad et al., 2021; Kildahl et al., 2017; Bakken et al., 2008).

In addition to psychopharmacological treatment, a crisis management plan, including the specific individual’s early warning signs of potential relapse, has been held as an important part of intervention to reduce the risk of relapse (Mohiuddin et al., 2011; Bäuml et al., 2006; Birchwood et al., 2000; Hogarty et al., 1991). In the present sample, 20 participants were provided with a crisis management plan, which included early warning signs, contact information for the patient’s general practitioner and specialist mental health services, and a plan for adapting to symptom sensitive communication with professional caregivers according to the patient’s current symptom load. Providing a crisis management plan involving professional caregivers may be even more important for autistic people with intellectual disabilities and schizophrenia than for other patients with schizophrenia, because these individuals may have difficulties recognizing and reporting early warning signs themselves. Training and supervision of community caregivers was provided for all 26 participants, highlighting the importance of collaboration between different service levels for these patients. Training and supervision of community caregivers may be particularly critical, because autistic people with intellectual disabilities are likely to require specific support in monitoring and managing symptoms, and this needs to be integrated in their community services.

For the sample as a whole, the symptom reductions achieved at the end of treatment (T2) remained at follow-up a year later (T3), and no significant increases in mental health symptoms or challenging behavior were found for any of the scales between T2 and T3. These findings provide a further indication that strategies used to reduce the risk of relapse in patients with schizophrenia in general, are helpful and applicable in autistic people with intellectual disabilities and co-occurring schizophrenia. Practical support has been described as an important aspect of schizophrenia treatment (Anderson et al., 1986). In the present sample, daily activities were adjusted following the diagnosis of schizophrenia for 24/26, a differentiated intervention plan was developed for 23/26, and demands were adjusted for 20/26. In a previous study on mental health nursing, providing increased practical support was described as aiding in symptom reduction for autistic individuals with intellectual disabilities and schizophrenia (Bakken et al., 2008). As the intensity of symptoms may vary during the course of treatment, continual adaptation of practical support may be necessary, and may be particularly critical for autistic people with intellectual disabilities who may have limited daily living skills prior to development of co-occurring schizophrenia. While not specifically reported in the current study, adapted provision of emotional support is important for these patients (Bakken et al., 2008), and constitutes an integral part of inpatient treatment in this population (Sommerstad et al., 2021; Bakken et al., 2008). Another important aspect of mental health nursing are communication skills (Bakken et al., 2008, 2017). Certain ways of communicating have been found to be helpful in symptom reduction and prevention of relapses: communication characterized by low levels of criticism, hostility and emotional over-involvement, and high levels of practical support (Anderson et al., 1986). Adapting mental health nursing to autistic individuals with intellectual disabilities and co-occurring schizophrenia may be challenging, and research is limited. Bakken et al. (2008) found it was a prerequisite for effective and therapeutic communication that ward staff had knowledge about the specific symptom presentations of schizophrenia in this population. This included the ability to establish joint attention with the patient, providing sufficiently adapted assistance with activities of daily living, and also responding meaningfully to the patient’s initiatives and providing emotional support (see also Sommerstad et al., 2021).

Family involvement has been held as an important aspect of providing mental health services for autistic people with intellectual disabilities (Chester et al., 2020; Bakken et al., 2017), and was reported for 18/26 participants. Because schizophrenia symptoms often persist over a longer period of time, especially negative symptoms (Færden, 2010), family involvement may be particularly critical in mental health services for patients with this diagnosis. While their input into evaluation of treatment interventions for mental health disorder has been held as critical (Rysstad et al., 2022), families often report difficulties accessing services, and difficulties in collaborating with services (Chester et al., 2020; Hellerud & Bakken, 2019). It is not clear why no family involvement was reported for almost a third of the sample. It is possible that some of the participants did not have family members who were involved, or alternatively, it may suggest that family involvement is an important area for continued development, in mental health services for autistic people with intellectual disabilities. For example, while utilization of these methods may be resource-demanding, adaptations of psychoeducational multifamily groups (McFarlane et al., 2003) have been described as being feasible and helpful for autistic people with intellectual disabilities and schizophrenia (Bakken et al., 2017).

### The Outcome Measures

There were significant reductions in symptom load and challenging behavior during treatment for the current sample, as measured on the PAC and the ABC. These significant changes were primarily found between admission/referral (T1) and the end of treatment (T2), and the reductions remained at follow-up, a year later (T3). On the PAC, the larger effect sizes were found for PAC psychosis and PAC general adjustment difficulties. On the ABC, the larger effect sizes were found for ABC hyperactivity/non-compliance and ABC inappropriate speech, scales that have recently been found to be associated with PAC psychosis in a larger, partly overlapping sample (Kildahl et al., 2023). Notably, the reduction in PAC psychosis was followed by a later reduction in PAC anxiety, which was significant from T1 to T3, suggesting that the reduction in anxiety symptoms for the current sample may have been secondary to reduction of psychotic symptoms. In addition, significant reductions were found for four of the five ABC scales, indicating that treatment of schizophrenia was associated with reductions in a range of challenging behaviors for these participants.

Findings from general research on schizophrenia indicate that negative symptoms may be more challenging to treat than positive symptoms, and outlast them (Færden, 2010). No significant change was found for ABC lethargy/social withdrawal, and the effect size for change in PAC depression was smaller than for the other PAC scales showing significant reduction. Together, this suggests that also in the current population, negative symptoms may be more challenging to treat, and may outlast positive symptoms.

To our knowledge, no previous longitudinal study has explored changes in mental health symptoms and challenging behavior during treatment in a sample of autistic individuals with intellectual disabilities and co-occurring schizophrenia. While further research is needed, these are promising results, indicating that treatment of schizophrenia is effective in autistic people with intellectual disabilities, and may lead to a specific reduction in psychotic symptoms.

### Strengths and Limitations

As for strengths, the study involved longitudinal data collection, including three time points, on a population that is often difficult to access and recruit for research. A comprehensive study protocol was followed, and assessments were multimethod and comprehensive (Bakken et al., 2023). The same standardized checklists were used across the three time points to assess changes in mental health disorder symptoms and challenging behavior. To date, there have been few studies of treatment of schizophrenia in autistic people with intellectual disabilities, beyond case studies or case series, and most studies have been focused on psychopharmacological treatment. While the current sample is small, we are not aware of previous studies including the same number of autistic people with intellectual disabilities and co-occurring schizophrenia.

Several limitations should be noted. Due to the lack of a comparison group, causal inferences are not possible, and the small sample size meant that further disentanglement of the potential effects of the specific interventions was not possible. Furthermore, the sample was recruited in the specific setting of specialized mental health services for people with intellectual disabilities in the Norwegian health care system. Interventions were only coded as yes/no, and more detailed information about the extent and content of the interventions is lacking, including how they were specifically adapted to each patient. Similarly, while information about psychopharmacological treatment was reported, more detailed information is lacking about what specific drugs and dosages were used. No systematic monitoring of side effects was included in the study protocol, nor were interventions that were not widely used when the study was designed (e.g., sensory therapy; Champagne, 2011). While the two checklists used appear to have adequate to good psychometric properties (Helverschou et al., 2021b; Halvorsen et al., 2019), and the PAC appears to have captured psychotic symptoms in the current sample (Bakken et al., 2023), the PAC is a screening checklist and not a diagnostic tool.

## Conclusions

A broad spectrum of interventions was provided to autistic individuals with intellectual disabilities and co-occurring schizophrenia, indicating that interventions developed for schizophrenia more generally are adaptable and applicable for this specific population. This further echoes previous recommendations that when diagnosed with co-occurring schizophrenia, autistic people with intellectual disabilities should be given access to the available range of interventions for this disorder. This includes interventions aiming to reduce the risk of relapses. However, adapting these interventions to the individual’s level of intellectual disability, communication skills, autism-related characteristics, and other relevant characteristics (e.g., sensory disabilities), is a clinically complex task and may require specific expertise in treatment of mental health disorder in this specific population. Furthermore, the treatment should include training and supervision of professional caregivers, in order to reduce the risk of relapse. Finally, as significant improvement was found across multiple aspects of mental health symptoms and challenging behavior, particularly for reported psychotic symptoms, the results of the current study indicate that treatment of schizophrenia is effective also in autistic people with intellectual disabilities.
