# Correction To: Schizophrenia in Autistic People with Intellectual Disabilities. Treatment and Interventions

**DOI:** 10.1007/s10803-024-06430-2

**Published:** 2024-07-11

**Authors:** Trine Lise Bakken, Jane Margrete Askeland Hellerud, Arvid Nikolai Kildahl, Ann Magritt Solheim-Inderberg, Oddbjørn Hove, Sissel Berge Helverschou

**Affiliations:** 1 Oslo University Hospital, Oslo, Norway; 2 Helse Fonna Health Trust, Haugesund, Norway

**Journal of Autism and Developmental Disorders**

10.1007/s10803-024-06286-6

In this article the author name “Jane Margrete Askeland Hellerud” was incorrectly written as “Jane Margrethe Askeland Hellerud”.

In this article, the order that the authors appeared in the author list was incorrect.

The original article has been corrected.

